# Scanning laser-induced endothelial injury: a standardized and reproducible thrombosis model for intravital microscopy

**DOI:** 10.1038/s41598-022-07892-z

**Published:** 2022-03-10

**Authors:** P. Larsson, V. Tarlac, T.-Y. Wang, T. Bonnard, C. E. Hagemeyer, J. R. Hamilton, R. L. Medcalf, S. H. Cody, N. Boknäs

**Affiliations:** 1 Australian Centre for Blood Diseases, Monash University, Melbourne, Australia; 2 Department of Molecular and Clinical Medicine, Sahlgrenska Academy, University of Gothenburg, Gothenburg, Sweden; 3 Monash Micro Imaging (MMI), Monash University, Melbourne, Australia; 4 INSERM UMR-S U1237, “Physiopathology and Imaging of Neurological Disorders” PhIND, Institute Blood and Brain @ Caen-Normandie (BB@C), UNICAEN, Normandie Université, 14000 Caen, France; 5 Department of Hematology, Linköping University, Linköping, Sweden; 6 Department of Biomedical and Clinical Sciences, Linköping University, Linköping, Sweden; 7 Hematologiska Kliniken Universitetssjukhuset Linköping, 581 85 Linköping, Sweden

**Keywords:** Experimental models of disease, Preclinical research

## Abstract

Vascular injury models are indispensable for studying thrombotic processes in vivo. Amongst the available methods for inducing thrombosis, laser-induced endothelial injury (LIEI) has several unique advantages. However, a lack of methodological standardization and expensive instrumentation remain significant problems decreasing reproducibility and impeding the adoption of LIEI in the wider scientific community. In this, study, we developed a standardized protocol for scanning laser-induced endothelial injury (scanning-LIEI) of murine mesenteric veins using the intrinsic 405 nm laser of a conventional laser scanning confocal microscope. We show that our model produces thrombi with prominent core–shell architectures and minimal radiation-related fluorescence artefacts. In comparison with previous methods, the scanning-LIEI model exhibits reduced experimental variability, enabling the demonstration of dose–response effects for anti-thrombotic drugs using small animal cohorts. Scanning-LIEI using the intrinsic 405 nm laser of a confocal laser scanning microscope represents a new method to induce standardized vascular injury with improved reproducibility of thrombus formation. The reduced need for instrument customisation and user experience means that this model could be more readily adopted in the research community.

## Introduction

Although in vitro experiments performed on isolated components of biological systems are important tools in preclinical medicine, there is a need to complement such reductionist approaches with more complex models incorporating the myriad of molecular interactions occurring in vivo^1,2^. As a growing number of underlying mechanisms for thrombotic disorders are gradually unveiled, correct modelling of the phenotypic effects of specific therapeutic interventions becomes impossible without reliable and detailed information from in vivo experiments^3,4^. For this reason, there is a growing need for sophisticated animal models capable of integrating the numerous overlapping and often redundant molecular interactions at play during thrombosis in vivo. For most scientific purposes, mouse models offer the most attractive alternative to meet this demand, both due to their practical advantages and the ever-increasing availability of conditional or constitutive genetically modified murine strains.

To enable a more detailed assessment of thrombotic mechanisms, murine thrombosis models need to be versatile, and enable researchers to measure many variables, such as platelet accumulation, fibrin deposition, procoagulant membrane exposure and platelet activation. Arguably the most significant step towards achieving such an ideal model was provided by Professor Bruce Furie’s group with the development of real-time fluorescence imaging of thrombus formation following pulse laser-induced endothelial injury (pulse-LIEI) to murine cremaster arterioles^5,6^. With this methodological breakthrough, it became possible to generate space- and time-resolved quantitative data of both platelet accumulation and fibrin deposition after vascular injury in vivo using confocal microscopy. Several other groups have since adopted and expanded this model to enable mechanistic studies of thrombus formation and to map the effects of interventions modulating platelet activation and signalling pathways in vivo^7–11^.

An important issue with animal disease models is reproducibility^12^. Poor reproducibility in animal research is multifactorial, with some of the most important barriers being: (1) a need for complicated and expensive instrumentation run by highly skilled operators; (2) insufficiency and ambiguity of methods; and (3) poorly standardized methods resulting in large experimental and user-to-user variation^13,14^. The pulse-LIEI model initially developed by Falati et al*.* in 2002^6^ is no exception in this regard, as successful use of the methodology requires considerable instrument customization, and the absence of a standardized experimental protocol puts high demands on operator expertise. These significant barriers have likely slowed down the dissemination of murine laser injury models of thrombosis into the scientific community. Additionally, in a recent study, Grover et al. demonstrated that established protocols for pulse-LIEI result in poorly reproducible thrombi with a high coefficient of variation (CV) even in the hands of highly experienced operators^15,16^.

We hypothesized that the reproducibility of LIEI could be improved by using the intrinsic components of a scanning laser confocal microscope system to deliver vessel injury (scanning-LIEI). The rationale behind our hypothesis was that: (1) the improved alignment resulting from identical optical pathways of the lasers used for imaging and LIEI would enable more accurate targeting of endothelial cells and reduce the irradiation dose necessary for injury generation, and that (2) the use of pre-defined and automatically executed injury protocols specified using the intrinsic software of the system would increase ease of use and reduce inter-operator variability. We show that the resulting model can be used for reproducible quantification platelet accumulation and fibrin deposition, with low expenditure of animals and minimal requirements for operator training and instrument customization.

## Materials and methods

### Intravital imaging platform

**Figure 1:**
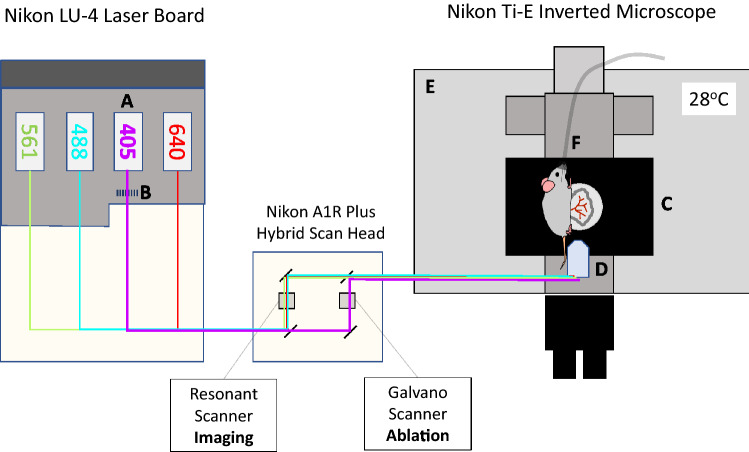
Schematic drawing showing the imaging platform used for the scanning-LIEI model. A standard Nikon A1R-plus dual scanner system was customised for live animal imaging and vessel ablation by the following modifications: The laser board was equipped with a 200 mW 405 nm CW solid-state laser (**A**) and the laser path was modified with an ND32 neutral density filter in a manual slider (**B**) allowing the 405 nm laser beam to be used at full power for laser ablation or attenuated for imaging. The system was further equipped with a Mad City labs Z-axis Piezo nanopositioner stage (**C**) for rapid imaging in the Z-dimension. The microscope was fitted with a Nikon CFI75 Apochromat 25XC W lens (**D**) with a high numerical aperture of 1.10, a long working distance of 2.0 mm and an adjustable ring to correct for depth-dependent spherical aberration. This lens is ideal for deep imaging of live specimens. The microscope stage was enclosed in a plexiglass box connected to a temperature control unit (**E**) and medical oxygen was supplied to the mouse via a nose cone (**F**).

The central components of our in vivo imaging platform are described in Fig. 1. The microscope platform used was a Nikon A1R + confocal (Tokyo, Japan) with a hybrid resonant and galvanometric scanner, mounted on a Nikon Ti-E motorized inverted microscope. A 200 mW 405 nm continuous wave solid-state laser (Coherent OBIS, Santa Clara, USA) was used for both ablation and imaging. Our choice of wavelength for photoablation was guided by the observation that low wavelengths reduced the scanning time needed to produce endothelial injury (data not shown). The ablation laser was housed in a laser combiner (Nikon LU-N4) modified with an ND32 neutral density filter (3.125% transmission) in a manual slider, so that the 405 nm laser could be attenuated when used for imaging, or used at full power for laser ablation. The laser combiner also housed a 488 nm, 516 nm and 640 nm Coherent OBIS solid-state lasers for imaging. The confocal microscope was equipped with two sensitive Gallium Arsenide Phosphide (GaAsP) and two conventional (bialkali) Photo Multiplier Tube (PMT) detectors. NIS Elements Software version 4.51.00 (Nikon Corp, Japan) was used to control the microscope platform.

The microscope objective was a dipping water immersion lens compatible with specimens mounted on coverslips (CFI75 Apo 25XC W, LWD, NA 1.10, CC 0–0.17, WD 2.0 mm, Nikon). The microscope was also equipped with a motorised Piezo Z-stage with high-speed Z-direction scanning capability (model NanoZ100-N, Mad City Labs, Madison, WI, US), enclosed in a temperature-controlled plexiglass box connected to heaters and a fuzzy logic temperature control unit (Clear State Solutions, Mount Waverley, Australia). For further information about the ablation laser used, please see supplementary “Methodological Considerations”.

### Antibodies and labels

The hybridoma clone 59D8, producing the fibrin antibody, was a kind gift from Professor Bruce Furie, Harvard Medical School, MA, USA. The antibody was produced by Monash Antibody Technologies Facility (Melbourne, Australia) and labelled with AlexaFluor-546 using the AF546 protein labelling kit (Life Technologies, A10237) according to the manufacturer’s instructions. Mouse platelets were labelled with an in vivo compatible modified antibody to mouse GPIbβ labelled with Dylight-649 (X-649 Emfret Analytics, Eiblestadt, Germany). FITC-labelled rat anti-mouse P-selectin (CD62P, clone RB40.34) and BV421-labelled rat anti-mouse CD31 was from BD Pharmingen (San Diego, USA). Pacific blue-labelled AnnexinV was from BioLegend (San Diego, USA).

### Anaesthetics and antithrombotic drugs

Sodium pentobarbitone (Lethabarb) was from Virbac, Australia. The direct thrombin inhibitor dabigatran (dabigatran etexilate, BIBR 953, SelleckChem) was dissolved to a stock concentration of 25 mg/ml in 5% v/v HCl in DMSO and stored at − 80 °C. The GPIIb/IIIa inhibitor eptifibatide (Integrillin, Merck, Sharpe and Dome) was stored at + 4 °C. Final concentrations of the drugs were prepared in sterile saline and injected i.v. (tail vein injection) at a maximum volume of 200 μl.

### Mice

To minimize the impact of light diffraction by perivascular adipose tissue, we only used young (typically 28–32 days of age, weight 16–21 g) male C57BL/6 mice for experiments. The mice were maintained on a 12 h light/dark cycle with food and water ad libitum. All procedures involving animals were approved by the local animal ethics committee of the Alfred Medical Research and Education Precinct (AMREP Ethics numbers E/1534/2015/B and E/1912/2019/M) and all procedures were performed in accordance with the AMREP ethics committee guidelines and recommendations. Results are reported in accordance with ARRIVE guidelines.

### Mouse preparation

Mice were anaesthetized with a 60–90 mg/kg intraperitoneal injection of sodium pentobarbitone then immediately placed on a heated mat and supplied with 100% O_2_ at 1–2 l/min via a nose cone. After a midline incision of the peritoneal cavity the mesentery was exteriorised onto a square coverslip (#1 Thermo Fisher Scientific) mounted on a stage insert. AlexaFluor-546 labelled anti-fibrin antibody (0.2 mg/kg) and Dylight-649 labelled anti-platelet (GPIbβ) antibody (0.1 mg/kg) were injected via the tail vein and allowed to circulate for at least 5 min before experiments. Pacific-Blue labelled Annexin V (0.02 mg/kg) was injected i.v. post-injury to avoid potential interference with thrombus formation. Mice were placed on the microscope stage in a temperature-controlled box set to 28 °C. Sodium pentobarbitone was administered to maintain anaesthesia during the experiment.

### Vessel selection and preparation

Mesenteric veins used for scanning-LIEI need to be carefully selected to ensure injury consistency. Important considerations for vessel selection are described in the supplementary “Methodological considerations” section. In brief, the following criteria were used: (1) Vessel type & size: mesenteric veins with an outer diameter range of 300–700 μm were included. (2) Vessel structure: Vessel sections with discernible fatty streaks, vascular branching or bifurcations were excluded. (3) Vessel wall movement: vessels exhibiting pulsative wall movements were excluded. For more details see supplementary “Methodological Considerations”.

### Scanning-LIEI optimisation

Standardization of laser scanning injuries was performed by the use of pre-defined protocols for vessel ablation using the “Photoactivation/Bleaching” feature of the NIS elements software. After manually localizing the endothelium using elastin autofluorescence as a point of reference, activation of this ablation protocol triggered automatized scanning of a circular region of interest (ROI) using the galvanometric scanner. The pre-defined protocol parameters that were found to influence injury characteristics were (1) laser targeting depth in the Z-dimension; (2) the scanning speed of the ablating galvanometric scanner (pixel dwell time) and (3) the dimensions of the ROI that defined the injury area. Systematic evaluation of these parameters allowed for injury optimization with regards to the size, structure, stability and reproducibility of the resulting thrombi.

### Image acquisition and analysis of fibrin and platelet volumes

Immediately after endothelial ablation with the galvanometric scanner, the resonant scanner was used to record time-lapse confocal Z-stack images. Images collected were of 512 × 512 pixels, scan speed of 30 fps, scanner zoom of 2 × (250 μm × 250 μm), bidirectional scanning, and 4 × line averaging. Emitted fluorescence in the 488, 561 and 647 channels was detected simultaneously with a pinhole size of 37 µm (1.1 airy units for the 640 nm laser). The piezo Z-stage was used to capture Z-stack images with a Z-axis step size of 1 µm and a Z-range of 80–100 µm to capture the entire height of the thrombus (time to acquire one Z-stack 10–13 s). The interval between Z-stacks was set to 60 s for the first 7 min following endothelial injury (growth phase), and then 120 s for an additional 10 min (maturation phase) to monitor the stability of the maturing thrombi over time. Thrombi were excluded from analysis if: (1) they contained auto-fluorescent or non-fluorescent regions (indicating tissue phototoxicity and “thrombus bleaching”, respectively), (2) the full thrombus height was not captured within the pre-set z-range (e.g. parts of the thrombus moving outside of the pre-set Z-range due to vessel relaxation), or (3) the vein moved during ablation resulting in an uneven injury. Less than 10% of recorded injuries were excluded from analysis due to these reasons (see Supplementary Table SI online). Image processing was performed with NIS Elements Advanced Research Software (Nikon). All analysed files were subjected to identical image processing steps and thresholding settings and processed in “batch mode” with minimal user interaction. Images were cropped, thresholded and processed using the smoothing and cleaning features of the software to separate the thrombus mass from free-flowing platelets, after which platelet and fibrin volumes or total fluorescent intensities were quantified. Prior to each injury, a z-stack image of the targeted area was acquired using identical laser settings to allow for background correction. Data was exported to Excel (Microsoft) and then graphed using GraphPad Prism v 8.0.2 (GraphPad, San Diego, CA, USA).

### Study design for dose–response effects of anti-thrombotic drugs

For each mouse, injuries were performed both before (untreated control) and after administration of anti-thrombotic drug or vehicle. Control and drug/vehicle-treated injuries were normally performed in the same vessel segment and subsequent injuries were performed approximately 500 µm upstream of previous injuries to minimise the influence of existing thrombi. For dose–response experiments with eptifibatide, each mouse received one bolus injection of either 10 mg/kg, 15 mg/kg or 20 mg/kg of the drug followed by one injury (due to the short half-life of the drug). The effect of eptifibatide on platelet accumulation was compared to non-treated injuries from the same group of mice. For dabigatran dose–response experiments, each mouse received one bolus injection of either 0.03 mg/kg, 0.1 mg/kg, 0.3 mg/kg or 1 mg/kg or vehicle control (volume matched acidified DMSO) followed by two or three injuries. The effect of the drug on fibrin accumulation was compared to vehicle control. The total number of animals used was 17 for the eptifibatide cohort displayed in Fig. 6. 40 mice were used to generate the control cohort data displayed in Fig. 5 as well as the dabigatran dose–response data displayed in Fig. 6. On average 3.6 injuries were performed in each mouse. For details of number of injuries generated in each cohort see Supplementary Table SII.

### Assessment of variability between users

In order to test whether a person with no previous experience of intravital thrombosis models could readily adopt the Scanning-LIEI technology, a scientist from a different laboratory at our institute (who had no specific experience of thrombosis models or our imaging platform) was invited to perform the experiments required for the eptifibatide data set. This person was provided a detailed written protocol and taught the surgical procedures associated with mesentery vein exteriorization. The microscope and Scanning-LIEI injury procedure was demonstrated by an experienced technician. Training included observing experiments performed on one animal and doing another one under supervision. The data set generated by this technician was then compared to an equivalent data set produced by the experienced technician.

### Statistics

Results are expressed as the mean of thresholded platelet (GPIbβ) or fibrin volume (µm^3^) +/− SEM unless otherwise specified in the figure legend. The number of injuries (and mice) utilised in each experimental group is outlined in each figure legend. Cumulative “area under the curve” data was calculated by adding the volumes or intensities for each time point of the time-lapse (doubling the values obtained for the 120 s-spaced time points to equalise the time-distribution throughout the experiment). Descriptive statistics and hypothesis testing were performed using GraphPad Prism. Grubbs Outlier test (alpha 0.05) was used to detect outliers. The D’Agostino-Pearson omnibus K2 test was used to test for normal distribution (alpha 0.05). The statistical significance of anti-thrombotic drug effects was evaluated on cumulative AUC data (ln-transformed in the case of log-normal distributed data) using one-way ANOVA with Dunnett’s multiple comparisons test. A *p* value of < 0.05 was considered statistically significant.

## Results

### Optimisation of a standardized laser scanning injury using the intrinsic laser of a conventional confocal microscopy platform

The following parameters were evaluated to optimize vessel wall injuries using the in-built 405 nm laser: (1) Laser ablation targeting (z-depth) (2) Laser scanning duration (pixel dwell time of the ablation laser) (3) Laser injury size (ROI area). The experimental readouts evaluated for injury optimisation were: thrombus size (fibrin volume and platelet volume), endothelial damage as indicated by annexin V binding (Supplementary Fig. S1 online), and thrombus structure (presence of imaging artefacts related to phototoxicity e.g. tissue autofluorescence or bleaching, Supplementary Fig. S1).

#### Laser-targeting depth

**Figure 2:**
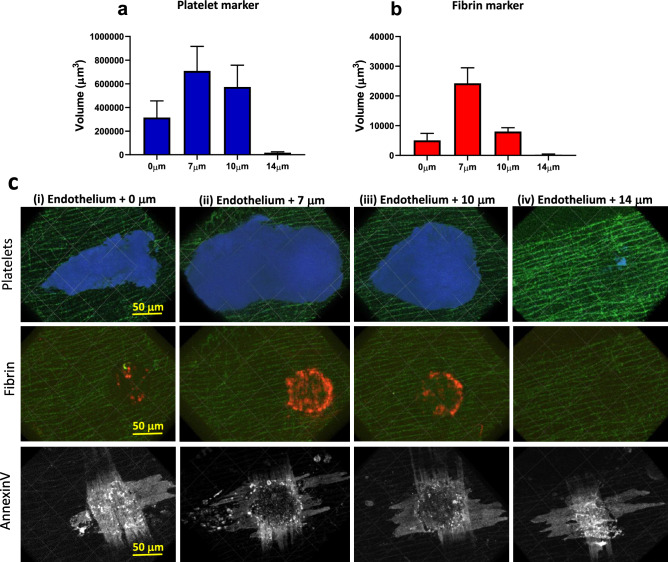
Optimisation of scanning-LIEI laser targeting depth. The scanning laser was targeted at the level of the endothelium (0 μm), 7 μm luminally of the endothelium (7 μm), 10 μm luminally of the endothelium (10 μm), or 14 μm luminally of the endothelium (14 μm). (**a**) Quantification of platelet volume 5 min after injury. (**b**) Quantification of fibrin volume 5 min after injury (n = 3–11 injuries from 3–5 mice). (**c**) Representative XY surface 3D reconstructions of thrombi (platelets blue, fibrin red), annexin V (white), and autofluorescent elastin layer (green). Blood flow is from right to left. Top row: platelet accumulation 5 min after injury. Middle row: Fibrin formation 5 min after injury. Bottom row: Annexin V binding > 30 min after injury. The laser was targeted (1) at the endothelium, (2) 7 μm luminally of the endothelium, (3) 10 μm luminally of the endothelium, or (4) 14 μm luminally of the endothelium. Note the strong annexin V binding to transverse smooth muscle cells and minimum fibrin formation after endothelium + 0 μm targeting. Also note the annexin V-positive endothelium without stable thrombus formation in the + 14 μm injury indicating that endothelial damage/activation alone is not sufficient for stable thrombus formation in this model. Scale bar and grid size 50 μm.

We found that the endothelial cell layer is located in the same optical section as the autofluorescent (488 nm) elastin fibres in the internal elastic membrane (IEM) of the vascular wall (Supplementary Fig. S1)^17^. Thus, IEM fluorescence was used as a proxy for the position of the endothelium and as a point of reference in the Z-axis when determining the targeting depth of the scanning laser injury. Unexpectedly, targeting the laser directly at the endothelial Z–layer yielded unstable thrombi and little fibrin formation (Fig. 2). We instead observed an incremental increase in thrombus size when the scanning laser was targeted further into the vessel lumen until reaching an optimal range of 7–10 μm luminally of the IEM. Using this targeting range, we observed substantial endothelial annexin V-binding (Fig. 2c lower panels), indicating correct targeting of the outermost cellular layers of the vascular wall, as well as fibrin formation and stable platelet accumulation in the injured region. Targeting the scanning laser > 10 μm luminally of the IEM resulted in minimal fibrin and platelet accumulation. A possible explanation for this unexpected optimal range of targeting depths could be the chromatic aberration of the objective lens at 405 nm^18^.

#### Laser scanning duration

**Figure 3:**
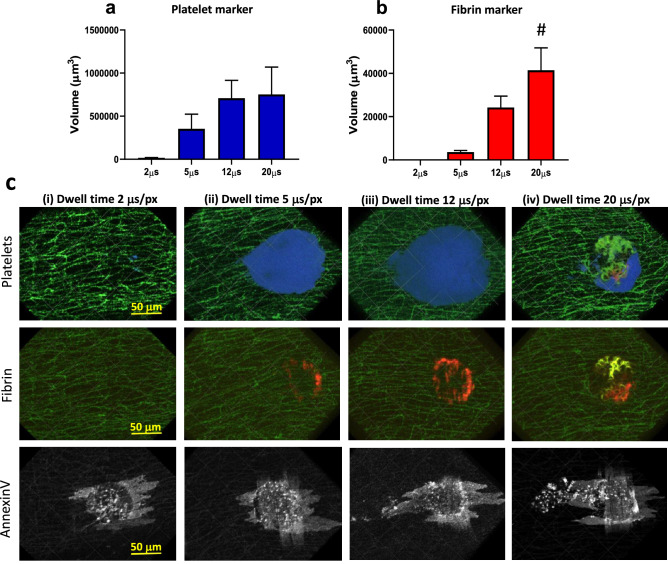
Optimisation of scanning-LIEI pixel dwell time (laser dose). The speed of the ablation scanner was varied to obtain different laser exposure times. (**a**) Quantification of platelet volume 5 min after injury at different scan speeds (2, 5, 12 or 20 μs/pixel). (**b**) Quantification of fibrin volume 5 min after injury at different scan speeds (2, 5, 12 or 20 μs/pixel) (n = 3–11 injuries from 3 to 5 mice). (**c**) Representative XY surface 3D reconstructions of thrombi (platelets blue, fibrin red), annexin V (white), and autofluorescent elastin layer (green). Blood flow is from right to left. Top row: platelet accumulation 5 min after injury. Middle row: Fibrin formation 5 min after injury. Bottom row: Annexin V binding > 30 min after injury. The speed of the ablation scanner was (1) 2 μs/pixel, (2) 5 μs/pixel, (3) 12 μs/pixel or (4) 20 μs/pixel. Note the autofluorescence of damaged tissue in the 488 nm (green) and 546 nm (red) channels (seen as yellow in the fibrin image) after 20 μs/pixel scan speed (see also Supplementary Fig. 1C) causing an artificially high signal in the fibrin (546 nm) channel (#). Also note the annexin V-positive endothelium in the 2 μs/px scan speed but lack of fibrin formation or platelet accumulation after 5 min indicating that endothelial damage/activation alone is not sufficient for stable thrombus formation in this model. Scale bar and grid size 50 μm.

One important model parameter determining the extent of tissue damage in LIEI is the radiation dose, i.e. the intensity of the ablation laser multiplied by the exposure time. For optimization of this parameter, we maintained the laser power at 100% in all experiments and calibrated the laser dose by varying laser exposure time. Using a laser scanning microscope, exposure time is defined by pixel dwell time (PDT), i.e. the time spent scanning each pixel of the ROI defining the coordinates of the laser injury. When evaluating a PDT range of 2–20 µs/px, we found an optimal PDT interval of 5–12 µs/px (Fig. 3), at which we consistently observed large thrombi without visible artefacts related to tissue phototoxicity. Short PDTs (< 5 µs/px) resulted in limited endothelial annexin V binding and very little fibrin deposition. The resulting thrombi were small and unstable, rendering them unsuitable for experimentation. At the other extreme, PDTs > 12 µs/px tended to produce autofluorescence in the 488 and 546 channels, with detrimental effects on subsequent analysis of fibrin accumulation (Fig. 3b 20 µs/px, c (iv)), as well as a high incidence of non-fluorescent structures (“black holes”) in parts of the image covering the region of the laser injury. The comparably long exposure times necessitated in our imaging-laser-based scanning-LIEI model confer an increased risk of photobleaching of fluorescent markers accumulating in the nascent thrombi during the ablation scan (“thrombus bleaching”). Using the lowest PDT in the optimal range (5 µs/px), the total time consumption for coverage of the entire ROI with the ablation laser was approximately 2 s in our system. At this time point, platelet and fibrin accumulation was still minimal, and a negligible amount of thrombus bleaching could be observed at the injury site.

#### Injury size

**Figure 4:**
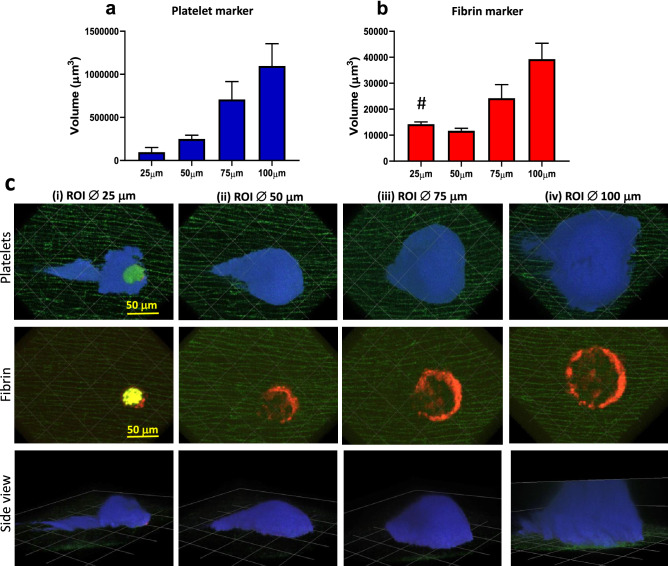
Optimisation of scanning-LIEI injury size (ROI diameter). Injuries were generated using different sized scanning ROIs. (**a**) Quantification of platelet volume 5 min after injury using different ROI diameter (25 μm, 50 μm, 75 μm, or 100 μm). (**b**) Quantification of fibrin volume 5 min after injury using different scanning ROI diameter (25 μm, 50 μm, 75 μm, or 100 μm) (n = 3–11 injuries from 3 to 5 mice). (**c**) Representative XY surface 3D reconstructions of thrombi (platelets blue, fibrin red), and autofluorescent elastin layer (green). Blood flow is from right to left. Top row: platelet accumulation 5 min after injury. Middle row: Fibrin formation 5 min after injury. Bottom row: Thrombus side view (YZ 3D surface reconstruction) 5 min after injury. The ROI diameter was (1) 25 μm (note the autofluorescence of damaged tissue in the 488 nm (green) and 546 nm (red) channels (seen as yellow in the fibrin image) causing an artificially high signal in the fibrin (564 nm) channel (#) (see also Supplementary Fig. 1C) (2) 50 μm (3) 75 μm or (4) 100 μm. Note the height of the thrombus exceeding our 100 um piezo stage Z-range limit when using the 100 um ROI. Scale bar and grid size 50 μm.

To optimize injury size, we evaluated thrombi generated from circular injuries with a diameter of 25 μm (area 491 μm^2^), 50 μm (1963 μm^2^), 75 μm (4416 μm^2^), or 100 μm (7850 μm^2^) (Fig. 4 and Supplementary Fig. S2 online). The most reproducible and stable thrombi were consistently achieved with ROI diameters of 50 or 75 μm. An injury diameter of 25 μm produced smaller thrombi and often resulted in notable tissue phototoxicity impacting the fibrin channel (Fig. 4b 25 μm, c (i)), whereas an ROI diameter of 100 μm frequently produced thrombi with heights exceeding our maximal imaging depth as defined by the range of the Piezo Z-stage (100 μm, Fig. 4c (iv) lower panel). In addition, we experienced difficulties finding suitably large veins that were compatible with the largest ROI injury diameter. This data indicated that the ROI diameters 50 and 75 μm yielded equivalent results when evaluated using the selection criteria stated above. However, as an increase in the dynamic range of the quantified variable (in this case thrombus platelet and fibrin content) could be considered advantageous from an analytical standpoint, we chose to use an ROI diameter of 75 μm in subsequent experiments.

### Scanning-laser induced endothelial injury to mesenteric veins produces reproducible platelet–fibrin thrombi with a prominent core–shell architecture

**Figure 5:**
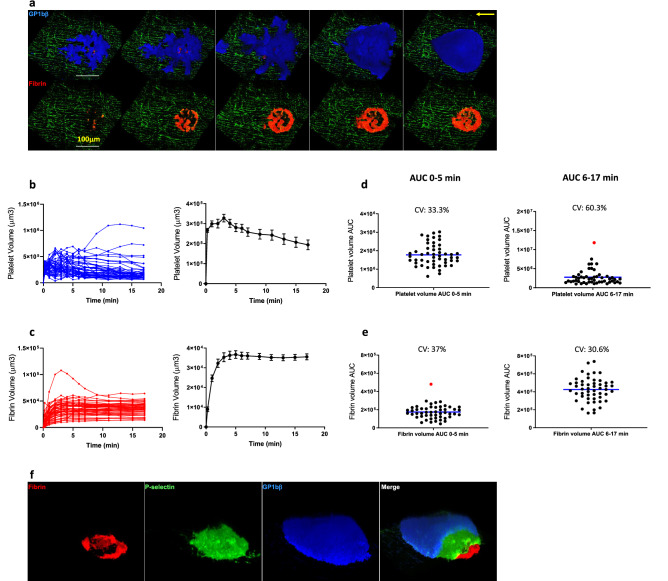
Scanning-LIEI causes dynamic platelet accumulation and results in reproducible thrombi with core–shell architecture. The formation of a thrombus after vessel injury was followed over time. (**a**) XY 3D reconstruction (surface rendering) of platelet (top panel) and fibrin (lower panel) structure at different time-points after scanning-LIEI (0.3 min, 1 min, 3 min, 5 min, 15 min). Platelets are shown in blue and fibrin in red. Scale bar = 100 um. See also Supplementary Video 1. (**b**) Platelet volume over time (0–17 min). Each injury is plotted in the left panel and mean (+/− SEM) is shown in the right panel. (**c**) Fibrin volume over time (0–17 min). Each injury is shown in the left panel and mean (+/− SEM) is shown in the right panel. (**d**) Scatter plot showing the distribution of cumulative platelet volume (area under the curve (AUC)) during the growth phase (0–5 min, left panel) and the maturation phase (6–17 min, right panel). (**e**) Scatter plot showing the distribution of cumulative fibrin volume (area under the curve (AUC)) during the growth phase (0–5 min, left panel) and the maturation phase (6–17 min, right panel). n = 51 injuries (37 mice). Data points determined to be outliers are shown in red. The mean and the Coefficient of variation (CV) is displayed in each scatter plot. (**f**) 3D reconstruction surface rendering of a representative thrombus 50 min after injury. The first three panels show the individual fluorescence channels (fibrin (red), P-selectin (green) and GpIb (blue)), and the fourth panel show a partial “peel-back” of each channel uncovering a core–shell structure with fibrin mainly present at the injury site, a core of P-selectin positive platelets and a shell of P-selectin negative platelets.

Having defined the optimal parameter ranges for our scanning-LIEI protocol, we characterized the dynamics and structure of the thrombi forming after scanning-LIEI in more detail. To this end, time-lapse experiments (51 injuries in 37 different mice) were performed to characterize thrombus formation using pre-defined optimal parameters for the scanning-laser induced endothelial injuries (Laser-targeting depth: 7 μm luminally from the endothelium, PDT: 5 µs/px, ROI diameter: 75 μm). Immediately following scanning-LIEI, we observed a very rapid expansion of thrombus platelet volume (0–1 min), followed by a plateau (1–5 min) and then a linear decline (6–17 min) (Fig. 5a, b and Supplementary Video 1). On average, a maximum total platelet volume of around 300,000 μm^3^ was reached after 2–4 min, equivalent to approximately 60–70,000 individual platelets^19^, with a gradual decrease down to 200,000 μm^3^ at the end of the experiments. The rate of fibrin accumulation was significantly lower, with a gradual decrease of fibrin growth rate during the first 5 min. The thrombus fibrin volume plateaued approx. 5 min after injury, reaching an average volume of 35 000 μm^3^, corresponding to 10% of the platelet volume, after which it remained largely constant during the remainder of the experiments, suggesting limited fibrinolytic activity during the examined time interval (Fig. 5a, c). Similar results were obtained when the analytical endpoints were changed from volume to total fluorescence intensities of the respective markers (Supplementary Fig. S3 online). Based on this distinct temporal pattern, we divided the data into two phases—a “growth phase” (0–5 min) and a “maturation phase” (6–17 min).

To estimate experimental variability, the coefficient of variation (CV) of the cumulative thrombus platelet and fibrin volumes (estimated as area under the curve, AUC) was calculated separately during the two phases of thrombus development. While platelet and fibrin volume measurements were found to be normally distributed during the growth phase, this only held true for fibrin volumes during the maturation phase, with platelet volumes displaying log-normal distribution during this time range. The CVs for the platelet and fibrin volumes were 33% and 37% during the growth phase, as compared to 60% and 31% during the maturation phase (Fig. 5d, e). These differences are likely a reflection of the differential spatial distribution of platelets and fibrin in the thrombus. Whereas fibrin deposition almost exclusively occurred in the stable environment of the thrombus core, platelets populated the highly dynamic peripheral regions wherein a considerable degree of variability could be observed during the maturation phase.

An increasing body of evidence indicate that thrombus formation in vivo is characterized by the establishment of a specific thrombus architecture, with fibrin at the base of the thrombus covered by a core of highly activated platelets and a shell of less activated platelets^10,20–22^. Using P-selectin expression as a marker for highly activated core platelets (alpha-granule release) we visualised the structure of the thrombi formed after scanning-LIEI (Fig. 5f). In line with previous findings, fibrin deposition was almost exclusively observed at the base of the injury, mainly associated with the area of the scanned endothelium. Occasionally, a diffuse fibrin signal would spread outside of the ROI injury area. Thrombi gradually developed a core of P-selectin-expressing platelets covered by a shell of platelets that did not express P-selectin. Thus, thrombi formed after scanning-LIEI are qualitatively similar to thrombi created by other LIEI models.

### Pharmacodynamic characterization of platelet inhibitors and anticoagulants using the scanning-laser-induced endothelial injury model

**Figure 6:**
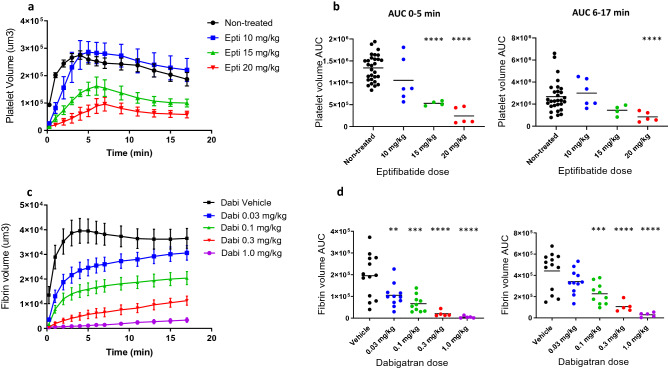
The scanning-LIEI model can be used to describe dose–response effects of anti-thrombotic drugs. Mice were treated with a bolus dose of the GPIIbIIIa inhibitor eptifibatide (Epti 10, 15, 20 mg/kg), vessel injuries were created using the scanning-LIEI protocol and platelet volume was measured at indicated time points. (**a**) Effects of eptifibatide on platelet accumulation (platelet volume) over time (mean +/− SEM). (**b**) Effect of eptifibatide on platelet volume AUC during the growth phase (0–5 min left panel) and maturation phase (6–17 min right panel). Number of injuries (mice) were as follows: Control = 29(15), Epti 10 mg/kg = 6(6), Epti 15 mg/kg = 4(4), Epti 20 mg/kg = 5(5). The mean of each group is displayed in the scatter plots. Due to the log-normal data distribution seen in the maturation phase this data set was log-transformed before subjected to one-way ANOVA. Mice were treated with a bolus dose of the direct thrombin inhibitor dabigatran (Dabi 0.03, 0.1, 0.3, 1 mg/kg) or vehicle and fibrin volume was measured at indicated time points. (**c**) Effect of dabigatran on fibrin volume over time (mean +/− SEM). (**d**) Effect of dabigatran on fibrin volume AUC during the growth phase (0–5 min left panel) and maturation phase (6–17 min right panel). Number of injuries (mice) were as follows: Vehicle = 13(6), Dabi 0.03 mg/kg = 11(4), Dabi 0.1 mg/kg = 10(4), Dabi 0.3 mg/kg = 5(2), Dabi 1 mg/kg = 5(2). The mean of each group is displayed in the scatter plots. **p < 0.01, ***p < 0.001, ****p < 0.0001.

Animal models of thrombosis play an important role when establishing the pharmacodynamic properties of antithrombotic drugs in vivo by measuring dose–response relationships. However, the relatively high degree of inter-experimental variability associated with available thrombosis models has made their use for such purposes particularly challenging, often necessitating large animal cohorts to compensate for the lack of experimental reproducibility. To investigate the utility of our scanning-LIEI model for the assessment of dose–response effects of both platelet inhibitors and anticoagulants, we measured scanning-LIEI-triggered thrombus formation after treatment with the GPIIbIIIa inhibitor eptifibatide (Integrilin) (10, 15, 20 mg/kg) and the direct thrombin inhibitor dabigatran (0.03, 0.1, 0.3, 1 mg/kg). As shown in Fig. 6a, eptifibatide caused a dose-dependent reduction of platelet recruitment to the thrombi immediately following scanning-LIEI (0–3 min). At the 10 mg/kg dose, platelet accumulation reached similar levels as in non-treated mice at later time points. However, with increased dosing (15 and 20 mg/kg) we observed a dose-dependent reduction of platelet volume AUC during both phases of the thrombus formation (Fig. 6b) which was most evident in the growth phase (left panel) but also remained at later time points for the 20 mg/kg dose (right panel). Paradoxically, eptifibatide had the opposite effect on thrombus fibrin deposition, with an approximately threefold increase in fibrin volume irrespective of dosing and phase (data not shown).

Similarly, a clear dose–response effect on fibrin growth rate and volume following scanning-LIEI could be demonstrated for the thrombin inhibitor dabigatran (Fig. 6c, d), with statistically significant effects at all the tested doses during the thrombus growth phase. Interestingly, a strong dose-dependent reduction in initial fibrin volume expansion was partially counterbalanced by a longer fibrin growth phase at the lowest dose (0.03 mg/kg) resulting in non-significant differences in AUC during the maturation phase. Although this effect was apparent also with higher dabigatran concentrations, prolonged fibrin volume expansion was not sufficient to compensate for the dramatic reduction of the fibrin growth rate, as reflected by significant reductions in fibrin volume AUC during the thrombus maturation phase with a dosing of 0.1 mg/kg, 0.3 mg/kg and 1.0 mg/kg dabigatran. At the two highest dose levels, dabigatran also significantly inhibited thrombus platelet accumulation in the growth phase, while only the highest dose resulted in significant thrombus platelet depletion in the maturation phase (data not shown).

## Discussion

A range of different vascular injury models have been developed for the study of thrombosis using intravital microscopy. The popularity of chemical (e.g. FeCl_3_), photochemical (e.g. Rose Bengal) and mechanical (e.g. needle puncture injury) injury methods have waxed and waned in the scientific community, but recently laser induced endothelial injury (LIEI) has become increasingly popular arguably due to its compatibility with fluorescence microscopy, allowing researchers to monitor the distributions of several different fluorescent probes throughout the volume of the developing thrombus^23^. The prevailing method for LIEI (herein called pulse-LIEI) involves ablation of murine cremaster arterioles with a powerful external pulsed nitrogen dye laser^6,8–10,24,25^. Although this approach has enjoyed widespread adoption globally, it suffers from a lack of methodological standardization. Using pulse-LIEI, injuries are generated by series of short laser pulses, the duration, number, and intensities of which must be adjusted manually for each individual experiment^5,23^. Apart from placing high demands on the expertise of the individual operator, this absence of a standardized and precisely defined protocol complicates independent validation and reproduction of experimental results. Additionally, a substantial amount of craftsmanship is required to avoid imperfect endothelial targeting with the high-energy external ablation laser, causing artefactual damage of surrounding vascular tissues. This methodological imprecision results in high degrees of experimental variability, even in the hands of very experienced operators^15^. Thus, large sample numbers are often needed for quantitative comparisons, making pulse-LIEI impractical for experimental studies wherein a low coefficient of variation is important, such as when studying dose–response relationships for new antithrombotic drugs.

The purpose of this study was to devise a simplified and standardized method for LIEI to improve the availability, ease of use and reproducibility of experimental models for fluorescence imaging of thrombotic processes in vivo. In the resulting scanning-LIEI model, a galvanometric laser scanning method is employed for endothelial injury, enabling the use of a continuous wave imaging laser for ablation which can be incorporated into conventional confocal microscope imaging systems. This allows for standardization of vessel injury with an automated, pre-defined injury protocol that controls the exact injury size and location as well as the dosing of the ablation laser. We show that once optimal injury conditions have been empirically determined, thrombi generated with scanning-LIEI are reproducible, stable and exhibit a characteristic core–shell architecture. To provide a comparative measure of experimental variability, we show that the coefficient of variation (CV) for platelet and fibrin AUC in thrombi generated with our model compares very favourably to those recently reported for pulse-LIEI^15^ (see also Supplementary Fig. S4 online where we plot our data on logarithmic scale and with the same number of steps on the Y axis as in the Grover et al. 2020 paper for direct comparison with data recently a generated by the Flaumenhaft group^15^). This improvement is likely the result of several contributing factors including precise control of injury size and radiation dose. Additionally, the relatively large injury size employed in our model is likely to make it less sensitive to small variations in injury size and depth. Further, we demonstrate that scanning-LIEI can be used to quantify dose–response effects of drugs inhibiting both platelet aggregation and coagulation. Due to its high degree of standardization, the scanning-LIEI method is comparatively easy to learn even without extensive in-person tutoring, with a low user-to-user variation. This is illustrated by the fact that a member of another research group at our institute was able to conduct experiments independently, with minimal training, and generate reproducible data sets within 3–4 weeks. The eptifibatide control data set of 29 injuries generated by this new operator (Fig. 6b) has a similar mean platelet volume AUC as the data in Fig. 5d as well as low variability (CV 23% for 0–5 min and 53% for 6–17 min). For a head-to-head comparison of the data sets generated by the two technicians see Supplemental Fig. S5 online. To facilitate adoption of our model by other groups, a supplemental document containing additional information is provided (Supplementary Note “Methodological Considerations”), and a detailed experimental protocol will be made available upon request.

As pointed out in a recent commentary on the subject of data variation in intravital microscopy thrombosis models^16^, there are several possible factors besides injury size that can contribute to variability in LIEI models, many of which are related to the specific characteristics of the individual blood vessel targeted for ablation. Using the scanning-LIEI model, this is particularly true, as the employment of a pre-defined scanning method for LIEI precludes any customization of laser injuries to compensate for individual differences in vessel characteristics. To address this issue, we provide a detailed description of the methodology used to identify suitable vessels in this study (see supplementary “Methodological Considerations”). One factor that can heavily influence injury quality in our model is the occurrence of pulsative vessel wall movements, as any movement along the Z-axis during LIEI scanning (duration approx. 2 s using our system) will move the ablation laser out of focus, dispersing the delivered laser dose over a thicker layer of the vessel wall. This, in combination with the increased diffraction caused by the thicker arterial vessel wall, leads to substantially longer dwell times being required for artery ablation and problems with photo-toxicity/bleaching of thrombus components accumulating at the injury site during ablation. This was the main technical consideration behind our choice to exclusively use veins for experimentation in this study.

As a core–shell thrombus architecture has been replicated using a range of injury models in different vascular beds (femoral artery^21^, cremaster arterioles^6,9,10^, saphenous vein^26^, jugular vein^22^, mesentery veins^27,28^, mesentery venules^20^), it is becoming increasingly evident that this structural feature is shared by thrombi forming in both the arterial and venous vasculature. As shown in Fig. 5f, results from our scanning-LIEI model are consistent with this general pattern. Thrombi generated with scanning-LIEI are thus more reminiscent of the “white”, platelet-rich, thrombi observed in arterial thrombi than the fibrin- and erythrocyte-rich thrombi found in deep venous thrombosis (reviewed in^29^). This finding suggests that our scanning-LIEI model can be used to estimate the effects of interventions targeting both the arterial and venous circulation, a notion supported by the significant dose–response effects demonstrated for both anticoagulants and platelet inhibitors in this study.

Apart from the improvements described above, the scanning-LIEI model described herein provides additional advantages that may prove valuable. Since thrombi generated with pulse-LIEI are very unstable over time, results from studies using this type of injury model typically only include data covering the first 2–5 min after vascular injury (e.g.^6,9,15,22,30^). Although such short time ranges can be adequate for modelling events occurring immediately after vascular injury, other important processes shaping the long-term fate of a thrombus are precluded from investigation. In this study, we provide statistically significant comparisons of treatment effects on thrombus platelet and fibrin content following scanning-LIEI during a time range of 0–17 min. This represents a considerable expansion, enabling the study of processes that are more prominent during the later stages of thrombus development, such as hydrodynamic stability and fibrinolysis. This temporal stability together with the prominent fibrin deposition in the core region of thrombi generated with scanning-LIEI strongly suggests that these injuries could be classified as “deep” according to the nomenclature developed in a previous study wherein immunohistochemistry was used to characterize laser-induced endothelial injuries^8^. Another interesting feature of scanning-LIEI is that the exact coordinates of laser injuries are recorded in the metadata of each image file, enabling exact mapping of the injury area in relation to the distribution of fluorescent probes in the thrombus. In ongoing studies, we aim to use this feature to provide a detailed and dynamic assessment of how thrombus heterogeneity is generated as a consequence of proximity to endothelial injury and direction of blood flow.

Given the artificial injury mechanism of LIEI models, their scientific merits are sometimes put into question. Such objections are perhaps best answered with the words of the statistician George E. P. Box: “All models are wrong, but some are useful”^31^. Considering the important contributions LIEI models continue to make to our understanding of thrombosis, their potential value should by now hardly be a matter of debate. However, by offering increased reproducibility, methodological standardization, and ease-of-use, we hope that scanning-LIEI will increase the usefulness of LIEI models for the scientific community.

## Supplementary Information

Supplementary Information 1.

Supplementary Information 2.

Supplementary Video 1.

Supplementary Video 2.

Supplementary Video 3.

Supplementary Video 4.
