# Supplementary material for: Scanning laser-induced endothelial injury: a standardized and reproducible thrombosis model for intravital microscopy
Source: Sci Rep. 2022 Mar 10;12:3955. doi: 10.1038/s41598-022-07892-z (PMC8913794; doi:10.1038/s41598-022-07892-z)
Supplement: Supplementary file 1 — Supplementary Information 1. [file 41598_2022_7892_MOESM1_ESM.pdf]

Scanning laser-induced endothelial injury – a standardized and reproducible thrombosis model for intravital microscopy

P. LARSSON, V. TARLAC, T-Y WANG, T. BONNARD, C.E. HAGEMEYER, J.R. HAMILTON, R.L. MEDCALF, S.H. CODY and N. BOKNÄS

Supplementary Tables

Supplementary Table SI. Summary of the number of injuries excluded from final analysis and reason for exclusion.

Group	*Total injuries*	*Excluded*	*Reason for exclusion from analysis*
Control	52	1	1 x Relaxation of vessel (bottom out of range)
Dabigatran/vehicle	46	4	2 x Relaxation of vessel (bottom out of range)<br>1 x Uneven injury<br>1 x Exceeding Piezo range
Rivaroxaban/vehicle	47	1	1 x Relaxation of vessel (bottom out of range)
Eptifibatide	52	9	5 x Relaxation of vessel (bottom out of range)<br>2 x Thrombus bleaching,<br>2 x Technical issues microscope
**Total:**	**197**	**15**	

Supplementary Table SII. Summary of the number of injuries performed in the two groups of mice (Control/NOAC group and Eptifibatide group)

Control/NOAC group (40 mice)	*Figure*	*Number of injuries*
Control (non-treated)	Fig 5b-e	52
Dabigatran dose-response	Fig 6c-d	46
Rivaroxaban dose-response	*(not reported in paper)*	47
	**Average inj/mouse**	**3.6**
Eptifibatide group (17 mice)		
Control	Fig 6a-b	36
Eptifibatide dose-response	Fig 6a-b	16
	**Average inj/mouse***	**3.06**

* Note that for the eptifibatide dose-response cohort only one eptifibatide-treated injury per mouse could be included in analysis due to rapid drug clearance from the circulation. Thus, a lower average number of injuries per mouse could be included in the analysis for this specific drug.

Supplementary figures

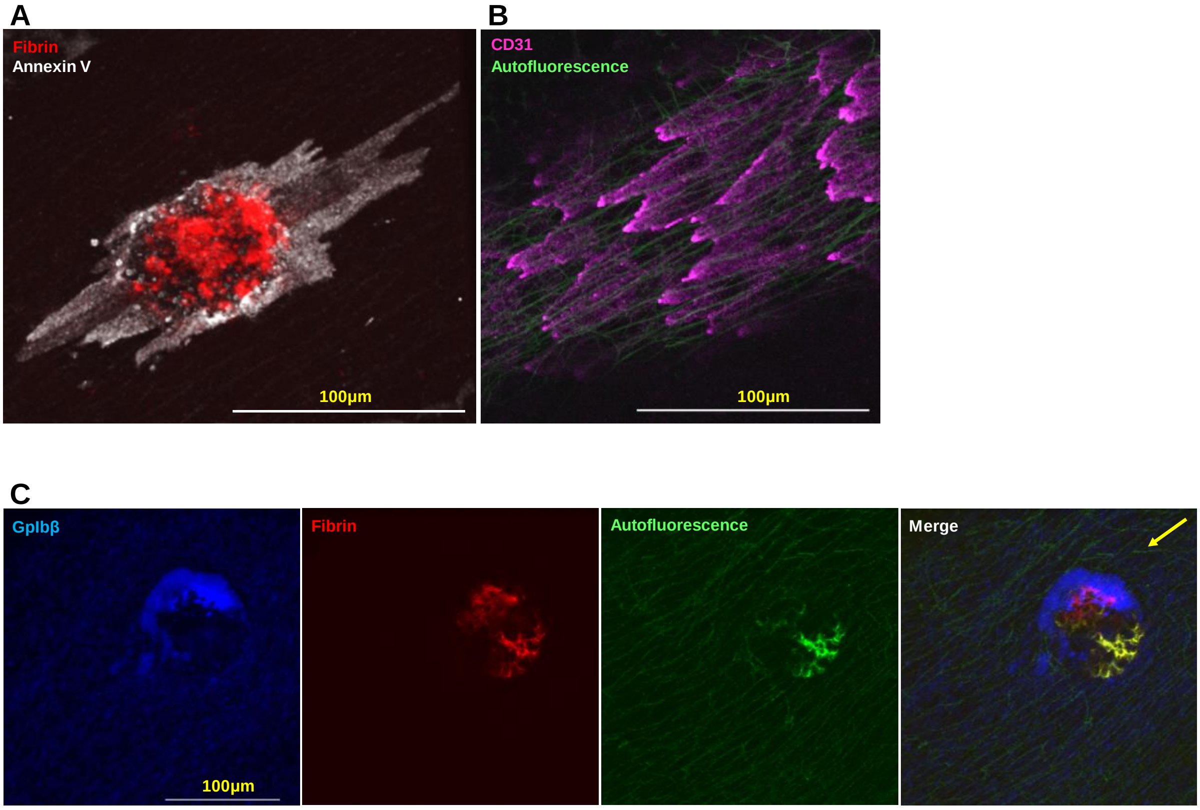

Supplementary Figure S1. A. Scanning-LIEI leads to endothelial damage and PS exposure. Images of mesentery vein endothelium after scanning-LIEI laser ablation. Annexin V-binding (phosphatidylserine-exposing) endothelial cells damaged by the scanning LIEI laser ablation are shown in white and fibrin in red. **B Endothelium is located in the same z-layer as autofluorescent fibrous extracellular matrix proteins.** BV-421-labelled anti-mouse CD31 was used to visualise the endothelium in mouse mesentery veins. High-resolution image of a 1 µm optical z-section showing that the endothelium (magenta) and autofluorescent extracellular matrix proteins (green) are visualised in the same optical section. Note the tile-like structure of the endothelium and the close z-proximity of the fibrous extracellular proteins and the endothelium. **C. Phototoxicity caused by excessive 405nm radiation dose.** Representative example of a thrombus forming after pixel dwell time > 12 µs/px. Excess radiation dose and/or poor targeting can result in the development of thrombi with areas of photobleaching and/or non-specific tissue autofluorescence. Platelets are shown in blue, fibrin in red, autofluorescence in green. The yellow arrow indicates the direction of blood flow. Note the black hole in the accumulating platelet mass ("thrombus bleaching", blue arrow) and the denatured tissue autofluorescence in the green (488mn) and red (546nm) channels (green arrows) seen as yellow in the merged image. Scale bars 100 µm.

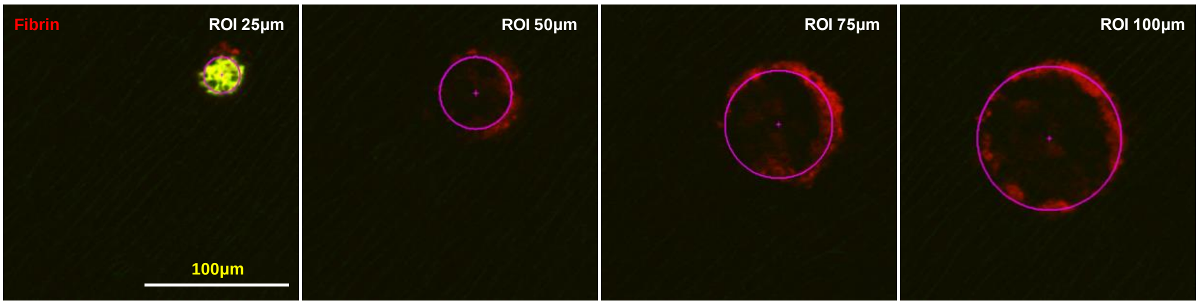

Supplementary Figure S2. Fibrin forms around the scanned/injured area. Fibrin formation 5 min after injury. Images of fibrin localisation (red) in relation to the position of the scanned ROI area (purple circle) when varying injury size (data presented in figure 4). Note how the fibrin signal is preferentially located just around the edges of the scanned region. The ROI diameter was 25µm (note the autofluorescence of damaged tissue in the 488nm (green) and 546nm (red) channels (seen as yellow in the fibrin image)), 50 µm, 75 µm or 100 µm.

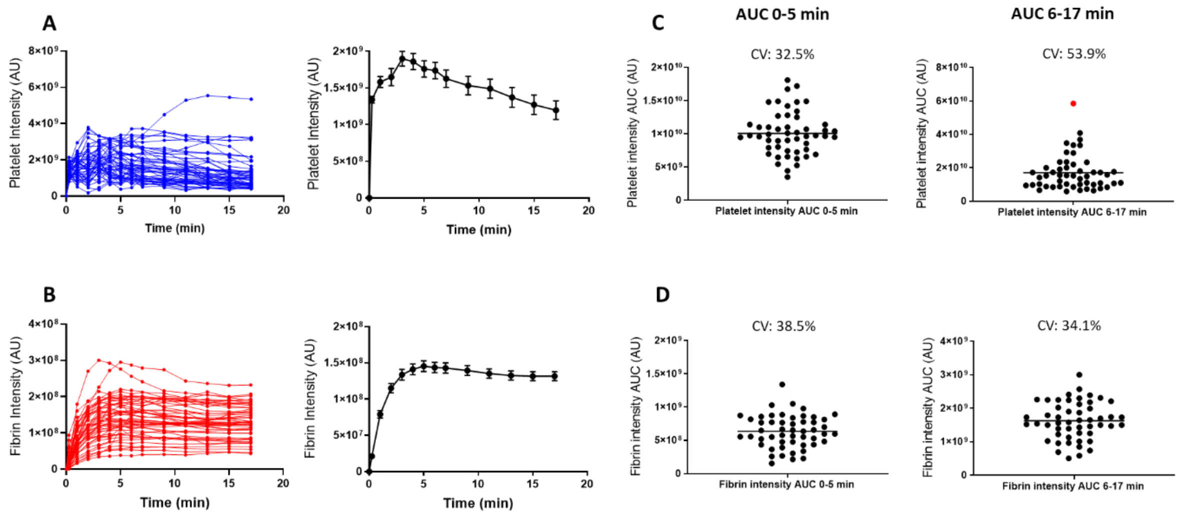

Supplementary Figure S3. Reproducible thrombus size after scanning-LIEI – total fluorescence intensity. Total fluorescence intensity data and AUC distribution for the same 51 thrombi as presented in figure 5B-E. **A)** Effects of eptifibatide on platelet fluorescence intensity over time (mean +/- SEM) **B)** Effect of dabigatran on fibrin fluorescence intensity over time. **C)** Platelet fluorescence intensity AUC 0-5 min (left panel) and AUC 6-17 min (right panel). **D)** Fibrin fluorescence intensity AUC 0-5 min (left panel) and AUC 6-17 min (right panel). Note the very similar temporal behaviour and distribution of AUC data compared to when measuring thrombus volume, indicating that the two readouts are comparable in this system.

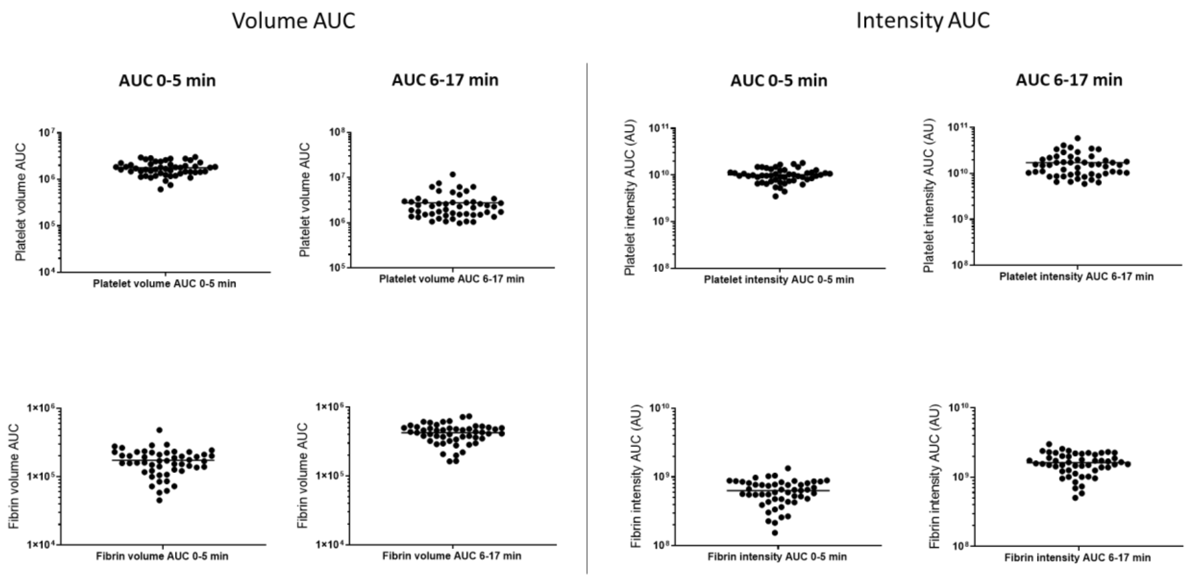

Supplementary Figure S4. Volume and intensity AUC data presented in Figure 5 plotted on logarithmic scale. Top panel: Platelet volume (left) and intensity (right). Lower panel: Fibrin volume (left) and intensity (right). All data is plotted using a logarithmic scale for comparison of data distribution with Grover et al 2020 (ref [15]). Note that the number of logarithmic steps on the Y-axis is the same as in Fig. 2, Fig. S1J and Fig. S3 in [15].

a

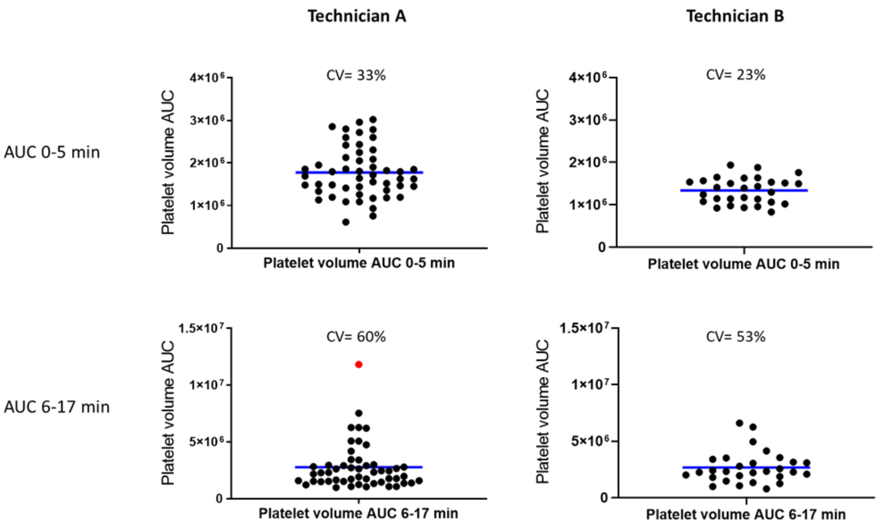

b

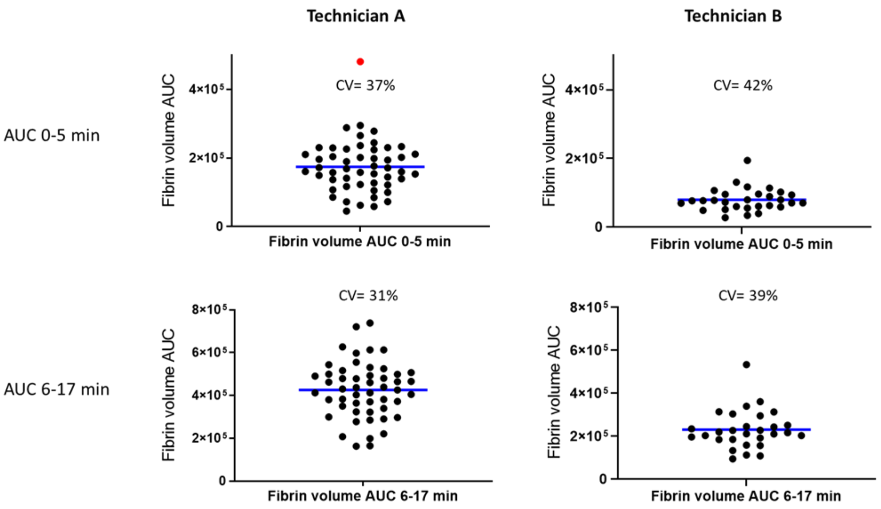

Supplementary Figure S5. Comparison of control injuries from scanning-LIEI generated by two technicians from different laboratories.

Scatter plots showing the distribution of cumulative platelet and fibrin volumes (area under the curve (AUC)) during the growth phase (0-5min) and the maturation phase (6-17 min) in scanning-LIEI control injuries generated by two technicians (Technician A and B). The mean and the Coefficient of variation (CV) is displayed in each scatter plot. **a)** Platelet volume. Note that the mean and distribution (relative standard deviation, CV) of the data generated by the two technicians are comparable. **b)** Fibrin volume. The distribution (relative standard deviation, CV) of the data sets are comparable between the two technicians, however the fibrin volumes are slightly lower in the cohort generated by Technician B. This is likely due to the reduced 561 nm laser and detector settings used to accommodate for the increased fibrin intensity in drug-treated mice (Technician A: Laser power 1%/gain 26. Technician B: Laser power 0.5%/gain 15) while identical thresholding settings were used for analysis. Note that these data sets are also displayed in Fig 5d and e (technician A, n=51) and in Fig 6b (Technician B, non-treated injuries n=29).

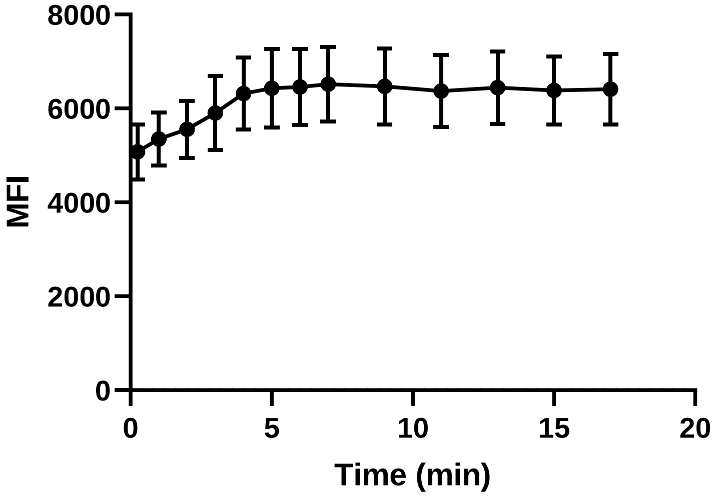

Supplementary Figure S6. Mean fluorescence intensity (MFI) of the X649 platelet marker over time. A stable MFI for the platelet marker (X649) in the thrombus over time indicate minimal imaging-induced bleaching of the fluorophore during the course of the experiment (n=51 injuries).
