# Supplementary material for: Scanning laser-induced endothelial injury: a standardized and reproducible thrombosis model for intravital microscopy
Source: Sci Rep. 2022 Mar 10;12:3955. doi: 10.1038/s41598-022-07892-z (PMC8913794; doi:10.1038/s41598-022-07892-z)
Supplement: Supplementary file 2 — Supplementary Information 2. [file 41598_2022_7892_MOESM2_ESM.pdf]

Supplementary Methodological Considerations for the scanning-LIEI model

Considerations - confocal imaging platform

We aimed to develop a laser injury model where a commercially available combined galvano/resonant scanning confocal microscope system could be used to both make a reproducible blood vessel injury and image the resulting thrombus in three dimensions with high temporal resolution. Unlike microscopes that require a third-party ablation system, the combined resonant and galvanometric scanners are perfectly aligned, enabling simple and accurate targeting of the endothelium. Ideally the resonant and galvano scanners should have the ability to scan the same field, and should be able to be used sequentially (or even simultaneously), switching between galvano and resonant modes in a short period of time (e.g. within seconds). The Nikon A1r+ confocal microscope was a suitable system for our needs. We initially custom equipped our system with a 50mW 405nm laser for ablation, but found that we required long exposure times (≥ 30 sec.) to cause vessel injury that was sufficient to cause a stable thrombus to form. Due to these lengthy ablation exposure times, the ablation also caused damage to platelet-associated fluorescent dyes since platelets started to adhere to the injured area during the course of the scan. So much so, that the resulting thrombus appeared to have a black hole at the injury site (thrombus "bleaching"). We therefore modified the system by installing a 200mW solid state 405nm laser equipped with a Neutral Density filter (ND32) to attenuate the 405nm laser for conventional imaging. Using this laser we were able to achieve sufficient vessel damage within a short enough time frame (2-5s) to avoid thrombus bleaching. Thus, based on this experience, we recommend using a 405nm laser of ≥ 200mW for endothelial ablation.

Considerations – selection of vessels

We chose to use mesenteric veins (MV) for scanning-LIEI model as: (i) MVs can easily be exteriorized onto a coverslip, enabling the use of an inverted microscope configuration (ii) the delicate vascular walls of MVs are optimally suited for use with the low energy internal laser source used in our model, as the energy loss of the laser due to tissue diffraction is proportional to vessel wall thickness; (iii) the relative absence of pulsative movements in the MVs in comparison with adjacent arteries would enable more accurate targeting of the endothelium; (iv) there are numerous mesenteric veins and they are of varying diameters allowing choice of vessels; and (iv) the relative ease of access would allow less experienced operators to perform experiments without extensive training.

While setting up the model we observed that even within the mesenteric vascular tree, qualities of the MV vessels such as wall thickness and composition, can have a large impact on injury severity and experimental reproducibility when using the scanning-LIEI model:

Absence of perivascular fat: Fat interferes with laser penetration and has detrimental effects on both laser ablation and imaging. Thus, it is important to consider the age and sex of mice that can be used for scanning-LIEI. Selecting mice by age and not weight can better control for the amount of perivascular deposits of adipose tissue in the mesentery. We have observed that age is a better predictor of mesenteric perivascular fat than weight in several strains of mice including the C57BL/6 mice used in this study. We recommend that only male mice of 4-5 weeks of age are used for scanning-

LIEI as the mesentery of these mice have less developed perivascular deposits of adipose tissue compared to females.

Size: The MVs selected need to be large enough to accommodate for the size of the scanning ROI used as the ROI needs to be positioned in a flat (without curvature) part of the vessel. The size of the vein also correlates to the thickness of the vessel wall which will determine the extent of the injury, since the ablation laser light is diffracted when going through tissue. A smaller vein has a thinner wall and thus the laser will be less diffracted and the injury more severe than in a larger vessel with a thicker vessel wall. Thus, reproducibility can be improved by selecting MVs of similar size, as we have shown by primarily selecting the primary branches of the ileal veins of the mesentery vascular tree.

Stability: MVs need to be immobile and relaxed while performing the scanning-LIEI ablation. Pulsatile vessel movements are often transmitted to surrounding structures such as veins by juxtaposed arteries in the mesenteric fascia, with detrimental effects on the quality of the LIEI and on subsequent imaging of the resulting thrombus (see ***supplementary video 2A*** for example of pumping MV and ***supplementary video 2B*** for example of stable MV). Further, after preparation of the mouse and exteriorisation of the mesentery, the vasculature needs time to stabilise and relax (approximately 10-15 min). If injuries are made on constricted veins, gradual muscle wall relaxation will cause progressive axial drift during the time-lapse acquisition, resulting in loss of full thrombus imaging capture as it moves out of the range covered by the pre-set Z-stack. The state of relaxation and movement of the vein can easily be determined by the appearance of the autofluorescent IEM elastin/collagen fibres in the vessel wall. Constriction is indicated by the close spacing of the IEM elastin/collagen fibres which increases upon relaxation (see ***Fig. 1A*** below for an image of a constricted MV and ***Fig. 1B*** for an image of the same gradually relaxed MV taken 5 min apart).

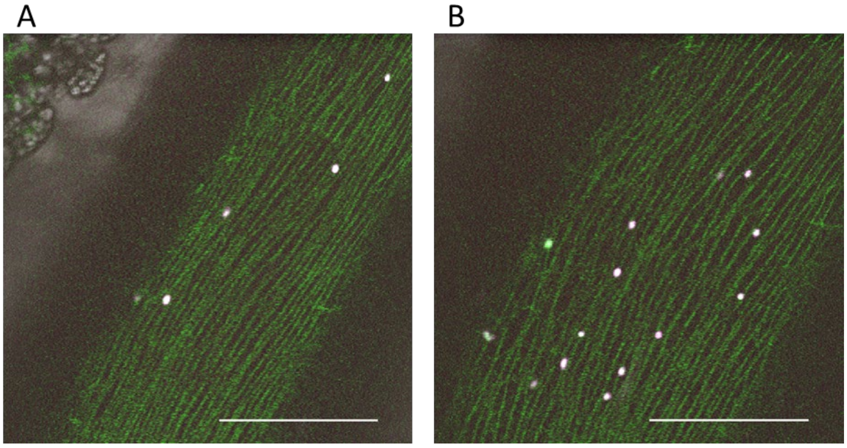

Figure 1 Use of the autofluorescent elastin layer to visualise vein constriction. A) MVs can be constricted from the preparation of the mesentery. Notice the tight spacing of the longitudinal green elastin fibres. **B)** Same MV segment 5 min later showing partial relaxation of the vessel. Note the increased spacing between the elastin fibres and the increased width of the vessel. Scale bar 100 µm.

Considerations - Imaging

The fluorophores used in the scanning-LIEI model need to be carefully chosen for optimal experimental set-up. In our system the 405nm imaging laser is also used for ablation, and the laser is switched from ablation to imaging mode by the manual insertion of an ND filter, a process that takes 2-5 s. Thus, if using the 405 laser for both ablation and imaging in the same experiment this has to be taken into

consideration as there will be a few seconds delay after ablation before imaging can commence. We therefore suggest avoiding the use of 405nm excited imaging fluorophores for any experiment that requires rapid imaging after thrombus initiation.

Autofluorescence is a naturally-occurring phenomenon observed in both animal and plant tissue. With the confocal settings used we could easily observe autofluorescence of the internal elastic membrane in the 488nm channel and found that these autofluorescent fibres and the endothelium are optically indistinguishable (Suppl. Fig S1). We routinely use these elastin fibres as a “point of reference” for laser targeting which result in reproducible laser injuries. Using a brightly 488-labelled platelet marker can interfere with visualising the autofluorescent elastin fibres and thus impact scanning-LIEI targeting. For example, when using a mouse model with GFP expressing platelets we were unable to distinguish the green autofluorescent elastin fibres used for targeting. Therefore, we suggest avoiding using a platelet marker in the 488 channel. We routinely use a far-red emitting platelet marker (Dylight-649) and green emitting fibrin marker (Alexa-546) as there is very little spectral overlap between these fluorophores allowing simultaneous acquisition to be used.
